# Supplementary material for: Four high-quality draft genome assemblies of the marine heterotrophic nanoflagellate Cafeteria roenbergensis
Source: Sci Data. 2020 Jan 21;7:29. doi: 10.1038/s41597-020-0363-4 (PMC6972860; doi:10.1038/s41597-020-0363-4)
Supplement: Supplementary file 1 — Supplementary Information [file 41597_2020_363_MOESM1_ESM.pdf]

Supplementary Information

Four high-quality draft genome assemblies of the marine heterotrophic nanoflagellate *Cafeteria roenbergensis*

Thomas Hackl[1,2], Roman Martin[3,4], Karina Barenhoff[1], Sarah Duponchel[1], Dominik Heider[3], Matthias G. Fischer[1]

Affiliations

1. Max Planck Institute for Medical Research, Department of Biomolecular Mechanisms, 69120 Heidelberg, Germany
2. Massachusetts Institute of Technology, Department of Civil and Environmental Engineering, Cambridge, MA-02139, USA
3. Philipps-University of Marburg, Department of Mathematics & Computer Science, 35032 Marburg, Germany
4. TUM Campus Straubing, Petersgasse 18, 94315 Straubing, Germany

corresponding author(s): Thomas Hackl (thackl@mit.edu), Matthias G. Fischer (Matthias.Fischer@mpimf-heidelberg.mpg.de)

Table of content

Supplementary Figure 1. Contamination screening of assembly CrE4-10P. 2

Supplementary Figure 2. Contamination screening of assembly CrBVI. 3

Supplementary Figure 3. Contamination screening of assembly CrCflag. 4

Supplementary Figure 4. Contamination screening of assembly CrRCC970-E3. 5

Supplementary Figure 5. Intron validation for the TATA-binding protein and 60S ribosomal protein genes by PCR and reverse-transcription PCR. 6

Supplementary Figure 6. Pairwise comparison of average nucleotide identities among strains. 7

For custom code and supplementary raw data see doi: 10.5281/zenodo.3551133

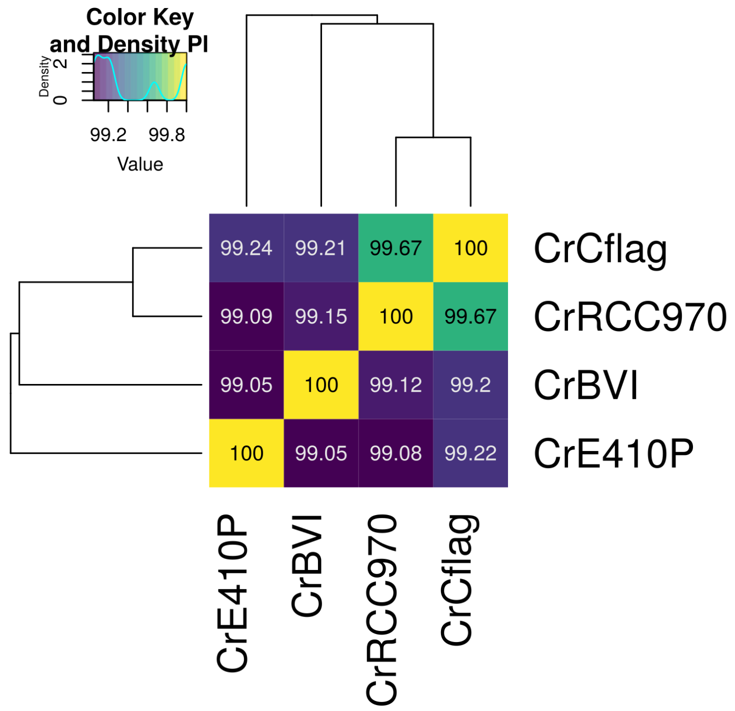

Supplementary Figure 1. Pairwise comparison of average nucleotide identities among strains. A heatmap showing the average nucleotide identities between all pairs of strains estimated using fastANI. Lighter colors indicate higher similarity. Rows and columns of the matrix are ordered according to the dendrograms shown above and left of the heatmap, and which were computed through

hierarchical clustering of the inferred distance matrix of the strains.

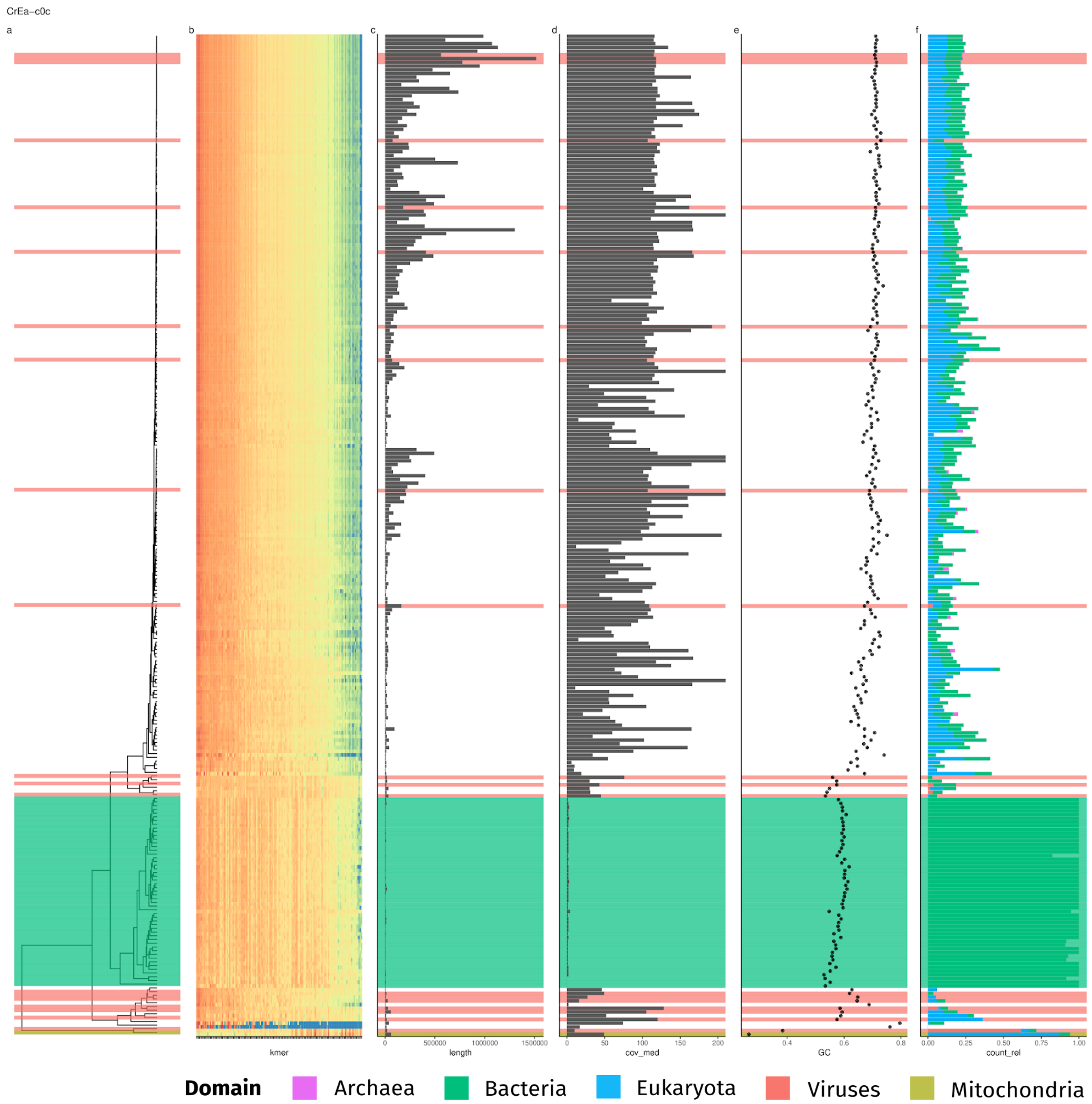

Supplementary Figure 2. **Contamination screening of assembly CrE4-10P.**

(**a**) Hierarchical clustering of contigs based on (**b**) scaled contig tetranucleotide frequencies. (**c**) Contig length. (**d**) Contig median coverage based on mapped Miseq reads. (**e**) Contig GC-content. (**f**) Distribution of taxonomic assignments at the domain level for 500 bp contig fragments. Colored bars present across all panels but (b) indicate sequences flagged as bacterial contamination (green), provirophage-containing (red) or mitochondrial (yellow).

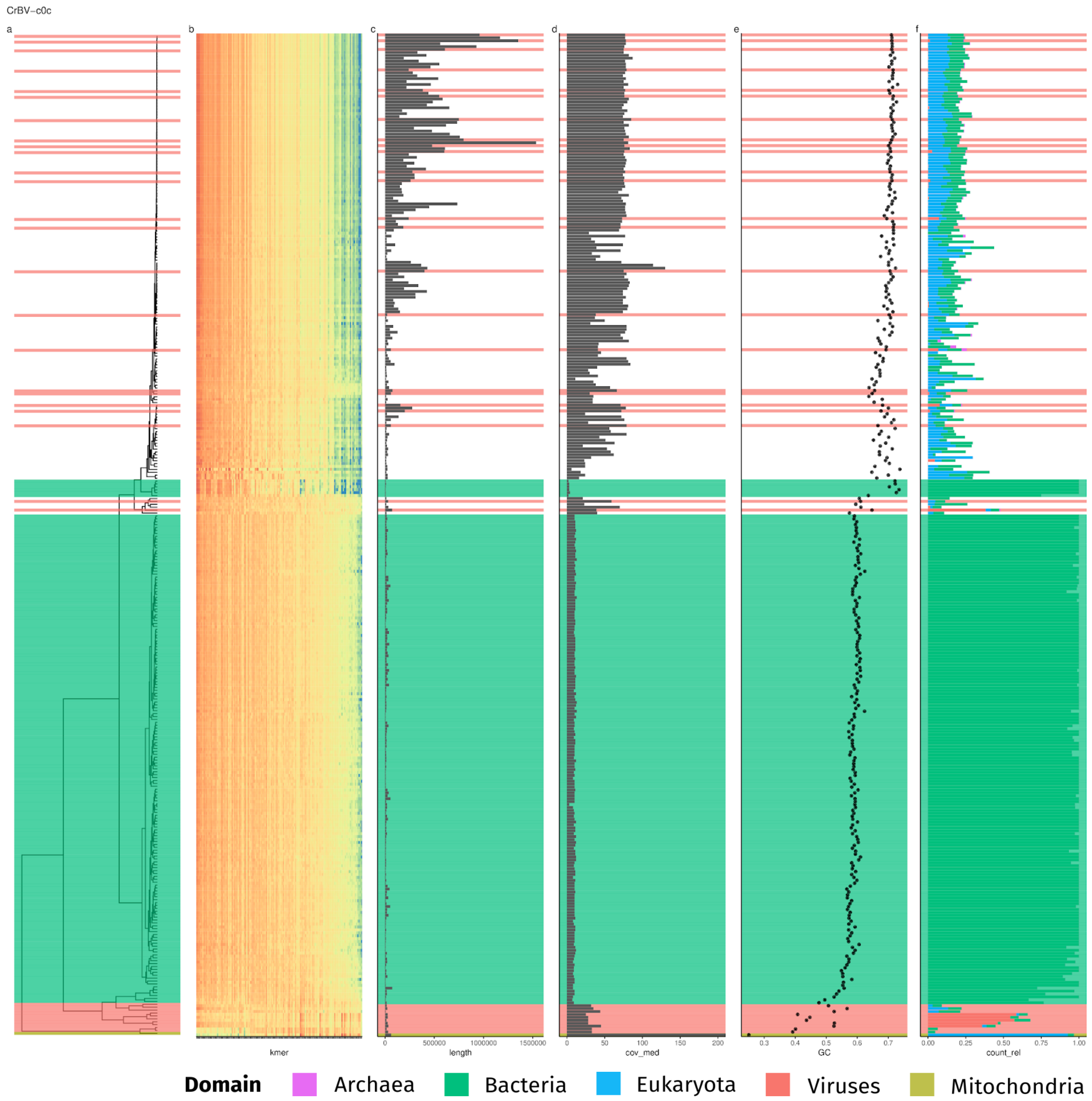

Supplementary Figure 3. Contamination screening of assembly CrBVI.

(**a**) Hierarchical clustering of contigs based on (**b**) scaled contig tetranucleotide frequencies. (**c**) Contig length. (**d**) Contig median coverage based on mapped Miseq reads. (**e**) Contig GC-content. (**f**) Distribution of taxonomic assignments at the domain level for 500 bp contig fragments. Colored bars present across all panels but (b) indicate sequences flagged as bacterial contamination (green), provirophage-containing (red) or mitochondrial (yellow).

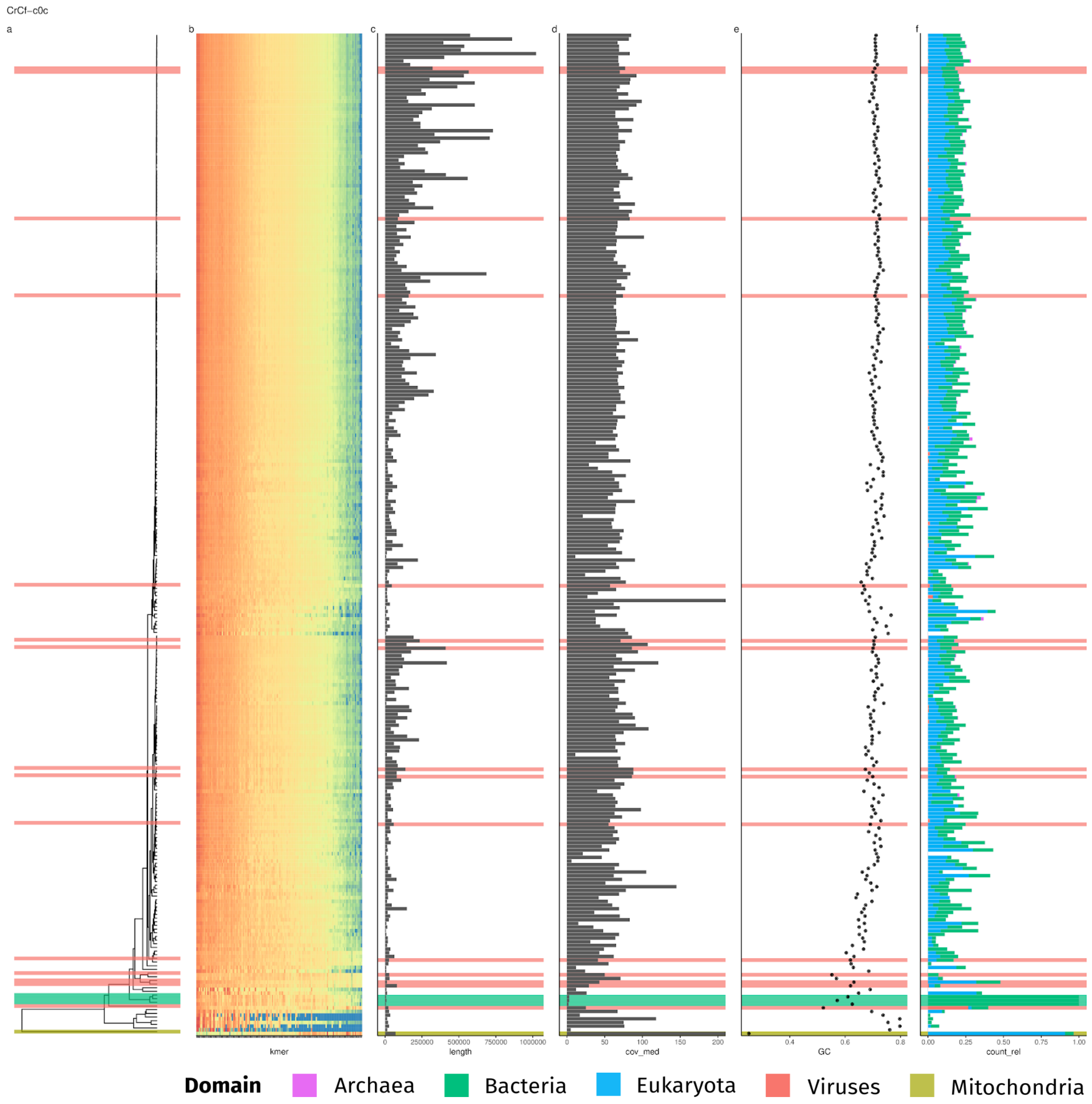

Supplementary Figure 4. Contamination screening of assembly CrCflag.

(**a**) Hierarchical clustering of contigs based on (**b**) scaled contig tetranucleotide frequencies. (**c**) Contig length. (**d**) Contig median coverage based on mapped Miseq reads. (**e**) Contig GC-content. (**f**) Distribution of taxonomic assignments at the domain level for 500 bp contig fragments. Colored bars present across all panels but (b) indicate sequences flagged as bacterial contamination (green), provirophage-containing (red) or mitochondrial (yellow).

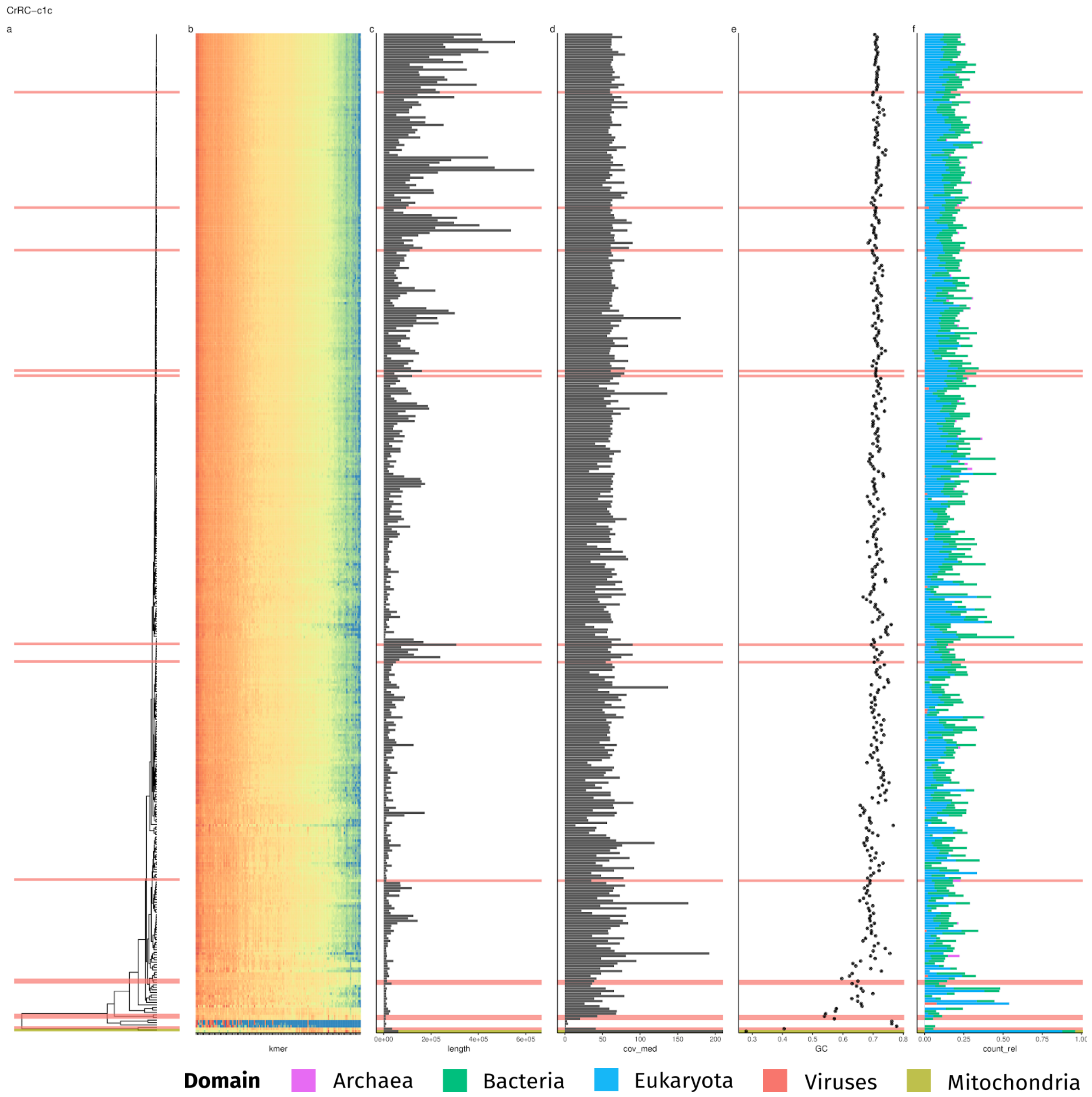

Supplementary Figure 5. Contamination screening of assembly CrRCC970-E3.
(**a**) Hierarchical clustering of contigs based on (**b**) scaled contig tetranucleotide frequencies. (**c**) Contig length. (**d**) Contig median coverage based on mapped Miseq reads. (**e**) Contig GC-content. (**f**) Distribution of taxonomic assignments at the domain level for 500 bp contig fragments. Colored bars present across all panels but (b) indicate sequences flagged as bacterial contamination (green), provirophage-containing (red) or mitochondrial (yellow).

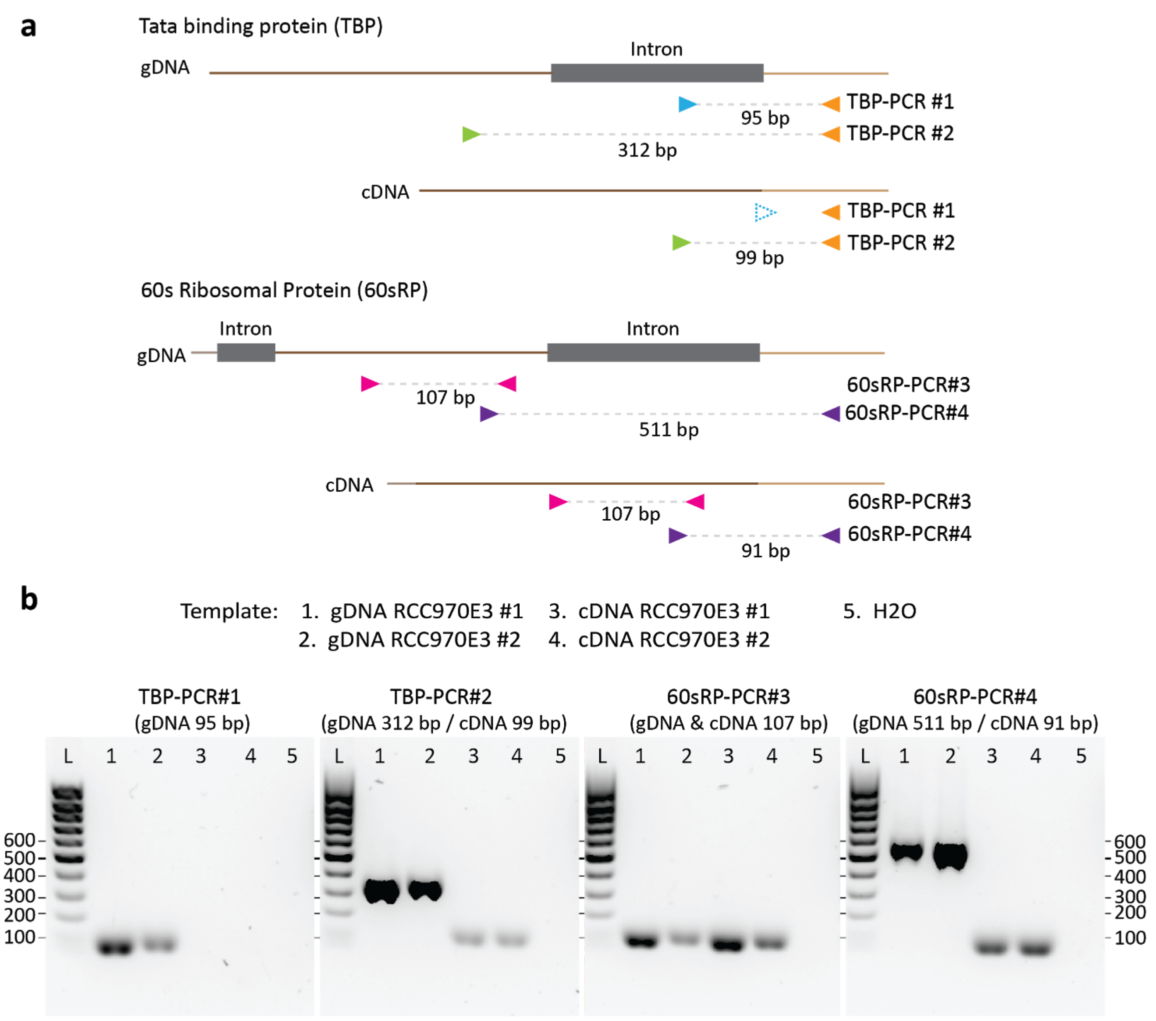

Supplementary Figure 6. Intron validation for the TATA-binding protein and 60S ribosomal protein genes by PCR and reverse-transcription PCR.

(**a**) PCR primer design to amplify the intronic region. (**b**) Gel images of the PCR products obtained from gDNA or cDNA of *C. roenbergensis* strain RCC970-E3. Each condition was analyzed in biological duplicates. The lanes are labeled according to the templates listed above. L, DNA size standard.
